# Nutrition in the Very Old

**DOI:** 10.3390/nu10030269

**Published:** 2018-02-27

**Authors:** Antoneta Granic, Nuno Mendonça, Tom R. Hill, Carol Jagger, Emma J. Stevenson, John C. Mathers, Avan A. Sayer

**Affiliations:** 1 Institute of Neuroscience, The Medical School, Newcastle University, Newcastle upon Tyne NE2 4HH, UK; antoneta.granic@newcastle.ac.uk; 2 NIHR Newcastle Biomedical Research Centre, Newcastle University and Newcastle upon Tyne Hospitals NHS Foundation Trust, Campus for Ageing and Vitality, Newcastle upon Tyne NE4 5PL, UK; 3 Newcastle University Institute for Ageing, Newcastle upon Tyne NE2 4AX, UK; n.m.p.mendonca@newcastle.ac.uk (N.M.); carol.jagger@newcastle.ac.uk (C.J.); john.mathers@newcastle.ac.uk (J.C.M.); 4 Institute for Health and Society, Newcastle University, Baddiley-Clark Building, Newcastle upon Tyne NE2 4AX, UK; 5 Human Nutrition Research Centre, Newcastle University, William Leech Building, Newcastle upon Tyne NE2 4HH, UK; tom.hill@newcastle.ac.uk (T.R.H.); emma.stevenson@newcastle.ac.uk (E.J.S.); 6 Institute of Cellular Medicine, Newcastle University, William Leech Building, Newcastle upon Tyne NE2 4HH, UK; 7 Academic Geriatric Medicine, Faculty of Medicine, University of Southampton, Southampton SO17 1BJ, UK

**Keywords:** the very old, nutrition, diet, dietary patterns, protein intake, malnutrition, physical functioning, the Newcastle 85+ Study, the LiLACS NZ, aged 80 and over

## Abstract

The population of older adults aged 85 years and over (the very old) is growing rapidly in many societies because of increases in life expectancy and reduced mortality at older ages. In 2016, 27.3 million very old adults were living in the European Union, and in the UK, 2.4% of the population (1.6 million) were aged 85 and over. Very old age is associated with increased risks of malnutrition, multimorbidity, and disability. Diet (nutrition) is a modifiable risk factor for multiple age-related conditions, including sarcopenia and functional decline. Dietary characteristics and nutrient intakes of the very old have been investigated in several European studies of ageing to better understand their nutritional requirements, which may differ from those in the young-old. However, there is a major gap in regard to evidence for the role of dietary patterns, protein, vitamin D and other nutrients for the maintenance of physical and cognitive functioning in later life. The Newcastle 85+ Study, UK and the Life and Living in Advanced Age, New Zealand are unique studies involving single birth cohorts which aim to assess health trajectories in very old adults and their biological, social and environmental influences, including nutrition. In this review, we have updated the latest findings in nutritional epidemiology with results from these studies, concentrating on the diet–physical functioning relationship.

## 1. Introduction

The extraordinary gain in human longevity and the rapid growth of the older population in both developed and developing nations [1] have been regarded among the greatest accomplishments of humanity, but they are also a cause for concern and a societal challenge [2]. As people enjoy longer lives, the main challenge will be to maximise the potential for these extra years to be ‘healthy years’ [3], and minimise the burden from disease, disability, and dependency [4]. Ageing is a multifaceted process, driven by a gradual and lifelong accumulation of molecular and cellular damage that leads to progressive loss of function in cells and tissues [5], increasing the risk of multiple diseases (morbidity) [4], disability and death. However, human ageing is a malleable process, as evidenced by the world’s changing age distribution and the rapid rise of older age groups (adults aged either 80 or 85 and over; the very old) in the last century [1]. Genetic and non-genetic factors, such as smoking, physical activity and diet, contribute to the heterogeneity in the ageing experience. There is substantial evidence from experimental and observational studies to support the roles of specific foods, dietary patterns, and nutrients in the prevention of chronic diseases and mortality [6,7,8] and in improving the quality of life with ageing [9].

However, there is a major gap in regard to evidence for relationships between diet (nutrition), health and functioning in very old adults who are at the highest risk of malnutrition [10], as well as the adverse health effects associated with nutrient deficiencies. In this review, we have updated and summarized recent epidemiological evidence from several European studies of ageing that have investigated diet and nutritional status in very old adults. We also report the latest findings from the Newcastle 85+ Study, United Kingdom (UK), and the Life and Living in Advanced Age: A Cohort Study in New Zealand (LiLACS NZ), the two on-going specialized cohorts involving the very old, with emphasis on the diet–physical functioning relationship.

This review is organized into five parts:

- The first part addresses (i) demographic transition and the increase of the very old population (Section 1.1); (ii) the importance of nutritional research in the very old (Section 1.2); and (iii) protein intake and muscle function as an example of the importance of nutritional research in very old adults (Section 1.2.1).
- The second part summarizes the current understanding of nutrition in the very old based on the insights from six European studies and two specialized cohorts of the very old that includes (i) what the very old eat and food sources of energy and nutrients (Section 2.1); (ii) nutritional statuses of the very old, with an example of micronutrient deficiency (Section 2.2); and (iii) nutritional needs of the very old, using the example of relationships between protein intake and vitamin D status and (objectively measured) physical functioning (Section 2.3).
- The third part updates the recent findings from the specialist cohorts dedicated to the very old, i.e., the Newcastle 85+ Study (Section 3.1, Section 3.2, Section 3.3 and Section 3.4) and the LiLACS NZ (Section 3.5, Section 3.6 and Section 3.7). This includes (i) the main characteristics of participants (Section 3.1) and dietary assessment (Section 3.1.1) in the Newcastle 85+ Study; (ii) the role of dietary patterns (Section 3.2) and nutritional biomarkers (serum vitamin D) (Section 3.3) in physical functioning in the very old; (iii) prevalence and determinants of low protein intake in the very old from the Newcastle 85+ Study (Section 3.4); (iv) the main characteristics of participants (Section 3.5) and dietary assessment (Section 3.5.1) in the LiLACS NZ; and (v) food contribution to macro (Section 3.6) and micronutrients (Section 3.7) in very old Māori and non-Māori adults.
- The fourth part addresses the challenges in establishing nutritional needs in the very old that are related to (i) nutritional assessments (Section 4.1); and (ii) heterogeneity in health in the very old (Section 4.2) and gives some suggestions for the future nutritional research in the very old.
- The fifth, concluding part highlights the main points discussed about the current state of knowledge of nutritional assessments, nutritional status and nutritional needs of very old adults.

### 1.1. Ageing Demographics: The Rise of the Very Old

The world population is rapidly ageing [11] with life expectancy at birth (LE0) having increased linearly by three months per annum over the last two centuries, with little sign of slowing down [12]. According to the recent US Census Bureau report [11], 617 million (8.5%) people in the world were aged 65 and over (older adults) in 2015, and numbers are estimated to reach 1.6 billion by 2050. Although the rate of population ageing differs across world regions and by economic wealth, developing countries in Asia and Latin America are experiencing a similar demographic transition to that of the Western world, and, by the middle of this century, almost 65% of the world’s older adults will live in Asia [11].

This remarkable gain in LE0 and growth of older age groups [11,12] is a result of a complex interplay between decreased fertility rates and reduced mortality—first in early life and more recently in late life. In particular, adults aged ≥ 65 comprise about 19.2% of the total population of the European Union (EU-28) [13]—a 2.4% increase over the last decade. Furthermore, the number of Europeans aged ≥ 80 has reached 27.3 million—an increase of 7 million. In 2015, 23 of the 28 member states of the EU-28 had an LE0 above 80 years and 20 had a life expectancy (LE) at age 65 above 20 years [14]. Eurostat reported that in 2016 in the EU the increases in LE at older ages have resulted in proportionately greater increases in the number of very old adults [15]. For example, in the UK, the Office for National Statistics (ONS) estimated that in 2016 there were 1.6 million very old adults (2.4% of the total population) [16], with numbers projected to rise to 3.2 million by 2041 [16,17].

Globally, the steady rise in LE and decrease in later life mortality make the very old (aged ≥ 85) the fastest growing age group of the Western world [12,18,19,20], and they are projected to increase by 351% from 2010 to 2050, compared to a 22% increase in older adults aged ≥ 65 [20].

Despite these gains in life expectancy, not all of the extra years have been healthy ones, with the increase in healthy life expectancy (HALE; number of years an individual can expect to live in a healthy condition) rising more slowly than LE0. The most recent figures from Eurostat show that, for the EU-28, LE at age 65 reached 21.2 years for women and 17.9 years for men in 2015, but, both men and women spent only 9.4 years healthy (free of activity limitation) [21]. Maintaining health in old age is of utmost importance, and, as diet is known to influence both health and mortality, dietary habits may contribute to the difference between LE and HALE.

Longitudinal cohort data are needed to improve understanding of the ageing process and its socioeconomic, psychological, and biological implications [22], as well as the societal demands for health and social services. There are a number of European ageing cohorts that include a large number of participants aged 50 and over, including the Survey of Health, Ageing and Retirement in Europe (SHARE) [23], the English Longitudinal Study of Ageing (ELSA) [24], the Irish Longitudinal Study on Ageing (TILDA) [25], and the Northern Ireland Cohort for the Longitudinal Study of Aging (NICOLA) [26]. However, the percentage of very old individuals in these studies is relatively small (e.g., only 3.4% of the total sample in the SHARE wave 1 in 2004/2005, and 6.5% in wave 6 in 2014/2015 were aged ≥ 85). There is, therefore, considerable uncertainty about how health and functioning change as we enter very old age, as well as whether risk factors for health earlier in life remain so in very late life. This stresses the importance of more specialized cohorts dedicated to the very old. Single year birth cohorts, such as the Newcastle 85+ Study [27], and the Life and Living in Advanced Age New Zealand: Te Puãwaitanga o Nga Tapuwae Kia Ora Tonu (LiLACS NZ) [28], are key to understanding specific research questions, including the role of nutrition, in the very old.

### 1.2. Why Is It Important to Research Nutrition in the Very Old?

Adequate intake of nutritious food is central to physical, psychological and social wellbeing at all life-stages, including in very old age. Diet is a major determinant of, and a modifiable lifestyle factor associated with, the development and management of a range of conditions and age-related diseases. These include ischaemic heart disease, stroke, atherosclerosis, type 2 diabetes, Alzheimer’s disease, obesity, and multiple cancers, which are frequently the leading causes of morbidity and death in Western societies and elsewhere [29,30].

In general, body mass, basal metabolic rate, and energy requirements decline with advancing age [31,32], which may compromise nutrient intake in older adults. Adherence to a healthy diet rich in nutrient-dense foods and high-quality diets (assessed by diet quality indices) is important at all stages of life but especially in later life where age-related diseases are more prevalent [33,34,35]. For example, there is evidence that an increase of 1–2 portions of fruit and vegetables per day may lower cardiovascular disease (CVD) risk by 30% [36]. A high consumption of saturated and trans-fat increases low density lipoprotein (LDL) cholesterol and total cholesterol, and decreases high density lipoprotein (HDL) cholesterol—risk factors for heart disease that may persist into very old age [37]. Eating a diet high in fat and sugar, and low in fruits, vegetables, and fibre is associated with several types of cancer, mainly in the gastrointestinal tract (e.g., mouth/pharynx and larynx, colon and pancreas) [37,38]. Several studies have indicated a possible protective role for omega-3 fatty acids in relation to CVD, dementia [39], and age-related loss of muscle mass [40,41].

Advanced age is associated with an increased risk of malnutrition, a nutritional state in which an imbalance, deficiency or excess of energy, macro (e.g., protein and fat) and micronutrients (e.g., vitamins and minerals) may cause measurable adverse health outcomes, as well as negative effects on tissues, organs, and body size, composition and function [42]. A recent systematic review and meta-analysis of the nutritional status of older adults has revealed that the prevalence of malnutrition, as assessed by the Mini Nutritional Assessment (MNA^®^), varies according to the health care setting and dependency level of individuals: from 3.1% for those living in the community, to 17.5% of those in care homes, and 28.7% in long-term care [43]. Micro and macronutrient deficiencies have also been repeatedly associated with ill-health and age-related diseases. For example, low vitamin D status may increase the risk of mortality [44], cognitive decline [45,46], muscle strength decline [47], CVD and low mood and depression [48]. A low status of B vitamins, especially folate, B12 and B6, has been found to be associated with an increased risk of stroke and cognitive decline [49,50]. Inadequate intake of dietary protein has been linked with poor muscle function and physical decline in older adults [51,52], including in the very old [53].

However, despite the increasing epidemiological evidence for the role of nutrition in the health and functioning of older adults, nutritional requirements of this age group are not well understood [37]. The evidence is particularly limited for the very old who may have different dietary requirements for energy, and macro and micronutrients to meet their health needs compared with, for example, the young-old (aged 65–74). The World Health Organization (WHO) has called for a revision of the current dietary (nutrient) recommendations and WHO guidelines for older adults [37] which can be used to aid national authorities to address the nutritional needs of their growing ageing population.

#### 1.2.1. Importance of Nutritional Research in the Very Old: Example of Low Protein Intake and Muscle Function

Older adults experience a gradual and progressive decline in skeletal muscle mass, strength and function with ageing (sarcopenia) [54,55], which intensifies in very old age [56,57], and increases the risk of falls, frailty, disability, loss of independence and death [58,59]. Depending on the operational definition, the age of participants, and the healthcare setting, the prevalence of sarcopenia varies from 1 to 33% [56]. The onset of sarcopenia is a central confounder for the health of an older person, because skeletal muscle accounts for 40% total body mass [60] and serves as a vital protein store and metabolic regulator [60,61], in addition to its primary functions related to posture, breathing, and mobility. The estimated direct healthcare costs of sarcopenia in older adults aged 60 and over in the US were $18.5 billion ($10.8 billion in men, and $7.7 billion in women) in 2000 [62]. Taken together, these facts emphasise the need for sustainable preventive measures aimed at preserving muscle health and function [56] in a rapidly ageing population.

The loss of muscle mass and strength associated with ageing is further accelerated by acute and chronic stressors, such as disease, physical inactivity [63], reduced mobility [64], and poor diet [51,52,56,65,66,67]. Adequate intake of dietary protein (providing essential amino acids; EAA) from animal and plant sources [68] in combination with resistance exercise (RE) [69,70,71] are recognised as key modifiable factors in promoting healthy muscle ageing and reducing physical decline [56,71,72]. Physiological intervention studies have shown that although the basal rates of muscle protein synthesis (MPS) in older muscle are comparable to those in young muscle [73,74], older adults experience a blunted response (anabolic resistance) after protein ingestion and/or exercise [75,76,77], especially to lower amounts of supplemental protein (or EAA) (<20 g (<10 g) post-exercise) [78] compared with young adults. Greater amounts of protein supplementation (>20 g) [51,76] and periodic feeding in combination with repeated bouts of RE [79] may result in greater muscle protein accretion and an increase in muscle mass [74,75,80], including in older adults diagnosed with sarcopenia [81] and frailty [82]. The age range of older participants in these intervention studies was ~65 to 80 (average age ~70–78) but studies with very old participants are lacking.

The current Recommended Dietary Allowance (RDA) for protein intake of 0.8 g/kg body weight/day (g/kg BW/day) [83] to support muscle health is the same for both younger and older adults and is based on a meta-analysis of a few nitrogen balance studies of short duration in mainly younger men [84]. A limited number of metabolic studies with adults aged 65–85 years, using amino acid oxidation as an index of protein adequacy (i.e., the indicator amino acid oxidation (IAAO) technique), have revealed higher RDA protein estimates of 1.2 g/kg BW/day for men and 1.3 g/kg BW/day for women [85], which were increased by a further 0.4 kg/kg BW/day in older men when based on fat-free mass (FFM) [85]. A study that used the same technique in women aged ≥ 80 yielded a minimum RDA protein estimate of 1.15 g/kg BW/day to support MPS and avoid muscle mass loss—30% more than the current RDA [86].

A number of recent expert position papers and reviews have summarized epidemiological evidence related to the optimal dietary protein intake for the maintenance of muscle mass and function in older adults and have argued for a higher protein intake of at least 1.0 to 1.5 g/kg BW/day [51,52,65,66,87,88,89]. To ensure effective simulation of MPS and to combat muscle wasting in older adults, it has been suggested that these higher protein intakes should be achieved through intake of ~25–30 g of protein per eating occasion across three main meals/day [51,52,65,66,78]. However, the debate continues about whether the optimal protein requirements differ not only across age, sex, body composition (e.g., FFM versus total BW; actual versus healthy BW [90]) and health status (e.g., renal impairment [91]), but also based on the long-term changes in clinical outcomes (e.g., lean body mass) and objective measures of physical functioning (e.g., grip strength (GS) and gait speed) [51,52,59,65,66,92,93]. The evidence from cohort studies of community-dwelling older adults aged ≥ 65 in regard to the impact of dietary protein on these outcomes needs further evaluation (e.g., [88,89], and a major gap exists in regard to evidence for very old, who are at the highest risk of functional impairments [94], sarcopenia [56,57], multimorbidity [95,96], and malnutrition [97,98,99,100].

As highlighted in the summary of the recent discussion of the European Society for Clinical and Economic Aspect of Osteoporosis, Osteoarthritis and Musculoskeletal Disease (ESCEO) working group (8 September 2016) [101], there is an increasing body of evidence for the role of nutrition in the management and prevention of sarcopenia (reviewed in [102,103,104,105,106,107]), muscle mass, strength and function in older adults (aged ≥ 65). The evidence to support the importance of protein [51,52,65,66,87,108] in combination with exercise and physical activity [51,52,65,66,87,88,89,107] in maintaining good muscle function during ageing is substantial but evidence is also emerging that other aspects of diet quality (e.g., features captured by dietary patterns) [109] and intakes of specific nutrients (e.g., vitamin D, antioxidant nutrients, and polyunsaturated fatty acids (PUFA)) may also be important for good muscle health in later life [101]. There is, however, a significant gap in epidemiological, physiological and intervention study evidence for the role of nutrition (diet, dietary patterns) in muscle health in very old adults. Further evaluations using, for example, pooled data from the several cohort studies and studies with dedicated cohorts of 85-year olds are essential for understanding their nutritional requirements for muscle function, which may differ from those observed in other age groups of older adults.

## 2. Current Understanding of Nutrition in the Very Old: Insights from the European Studies and Specialized Cohorts of the Very Old

Obtaining accurate quantitative assessments of food choices and estimates of habitual intakes of foods and nutrients is difficult at all life stages [110] but doing so in the very old poses several challenges for the researchers, including participants’ cognitive and physical impairments, reliance on proxy reporting (by a carer or spouse) for those who lack capacity, limited involvement of some participants in food shopping and preparation, and researchers’ consideration for participants’ burden during the interview. In the future, advances in dietary assessment technologies, including the use of urinary metabolomics, may provide objective estimates of food intake without the need for subjective dietary reporting [111]. Until then, dietary assessment methods intended for use in this age group should be validated to provide accurate estimates of intakes of foods, energy and nutrients in the very old [112].

### 2.1. What the Very Old Eat: Food Sources for Energy and Nutrients

Because of the challenges that dietary assessment presents in the very old, it is not surprising that dietary intake data in this age group are rare. Only a few population based studies dedicated to those aged ≥ 85 have assessed their dietary intake (e.g., the Newcastle 85+ and LiLACS NZ), and some representative and non-representative studies have attempted to measure energy and nutrient intake of ≥80 or ≥85-year olds in Europe. Table 1 summarizes the main characteristics, study population, and dietary assessments used in these studies.

Table 2 shows the energy and nutrient intakes in the very old by sex from seven European studies and the LiLACS non-Māori sub-cohort (i.e., European descendants). Comparison of nutrient intakes between these studies should be done cautiously because of the differences in the dietary assessment methods, data collection period, sample size, food composition tables used and nutrient definitions. Specifically, of all studies with significant numbers of adults aged ≥ 80 or ≥85, three used 24-h recalls, two used FFQs and three used different forms of dietary records. EPIC-Oxford had the largest number of adults aged ≥ 80 (*n* = 1283) of the eight studies.

Men and women from the German Nutrition Survey and EPIC-Oxford had the highest energy intakes (>9.25 and 8.0 MJ/d for men and women, respectively). Overall, 41–50% of the energy intake came from carbohydrates, 31–40% from fat and 14–16% from protein. In the Newcastle 85+ Study, participants had a median energy intake of 6.65 MJ, where 46.8%, 36.8% and 15.7% were from carbohydrate, fat and protein, respectively [97], which was comparable with those reported in the LiLACS NZ study (non-Māori) [119]. Dietary fibre intake varied considerably between country and study and depended largely on the dietary assessment method (FFQ versus 24-h recall) and analysis method (Englyst or the Association of Official Agricultural Chemists (AOAC)).

Vitamin and mineral intakes in the EPIC-Oxford were, on average, higher than in other studies, which may be attributed to the choice of dietary assessment and participant’s age. In the Newcastle 85+ Study, median vitamin D, magnesium, potassium and selenium intakes were 2.0 (IQR: 1.2–6.5) µg/day, 215 (IQR: 166–266) mg/day, 2477 (IQR: 1890–3023) mg/day and 39.0 (IQR: 27.3–55.5) µg/day, respectively [98]. In the LiLACS NZ study (non-Māori), the intakes of these micronutrients were 3.7 (IQR: 2.3–5.9) µg/day, 258 (IQR: 214–321) mg/day, 2755 (IQR: 2243–3285) mg/day, and 39.5 (IQR: 27.0–56.5) µg/day, respectively [120]. Higher dietary vitamin D values in the LiLACS study may be attributed to differences in the food sources between the two studies (e.g., milk, butter/margarine and fish/sea food were the top thee sources of vitamin D in the LiLACS, whereas fish/sea food, cereal/cereal products and meat/meat dishes were the main sources in the Newcastle 85+ Study). Vitamin D fortified dairy products are more commonly available in New Zealand than in the UK (New Zealand Ministry of Health, 2018). Diets in both cohorts provided very similar intakes of folate (in men), and vitamin B12 (Table 2).

In the Newcastle 85+ Study, cereals and cereals products (CCP) and non-alcoholic beverages were the only food groups consumed by the whole cohort, while oils and fat spreads, vegetables, dairy, potatoes, sugar, preserves and confectionery, and fruit were consumed by at least 75% of the participants [97]. CCP, especially flour and breakfast cereals, were the top contributors to intakes of energy, most macronutrients and some micronutrients (carbohydrate, non-milk extrinsic sugars, fibre, fat, folate, iron and selenium), followed by meat and meat products [97,98]. In the LiLACS NZ (non-Māori), bread, butter/margarine, fruits, milk, and cereal (grains) contributed to ≥75% of energy [119]. Bread, fruits, and grains were the main sources of carbohydrates; and meats and milk were the top sources of protein in women and men, respectively. For most micronutrients (e.g., folate, vitamin B12, iron, magnesium, vitamin D and calcium), the main sources included cereal, bread, milk, vegetables, and fish [120]. About 35% of non-Māori participants in the LiLACS NZ study got their vitamin A intake from vegetables [120], whilst the main source (40%) of the vitamin in the Newcastle 85+ Study was meats and meat products [98]. Similarly, for more than 23% of participants in the LiLACS NZ study, milk was the main source of vitamin B12 [120], whereas for over 50% of participants in the Newcastle 85+ Study, the main source of vitamin B12 was meats/meat products [98]. The contribution of food groups to nutritional intake in the NDNS for non-institutionalised ≥ 85 years was similar to that of the Newcastle 85+ Study. However, more vitamin B12 (29% versus 13%), calcium (54% versus 31%) and potassium (20% versus 9%) came from dairy in the NDNS [113] than in the Newcastle 85+ Study [98]. The food sources of vitamin D were considerably different between the studies with fish and seafood dishes, making a lower contribution to intake (17% versus 34%), while fat spreads made a higher contribution (23% versus 8%) in the NDNS [113] than in the Newcastle 85+ [98].

In summary, there appear to be considerable differences in intakes of energy and nutrients across European studies that have included the very old, perhaps due to methodological factors, such as dietary assessment methodology, nutrient definitions and food composition tables, in addition to any true population intake differences. Except for a few nutrients (e.g., vitamin D and folate) the Newcastle 85+ Study and LiLACS NZ, had comparable intakes; these two specialized cohort studies of the very old used the same dietary assessment. However, the preferred dietary sources of several nutrients also varied across these two studies, notably, milk was the main source of protein in the LiLACS NZ (women) compared to meat/meat products in the Newcastle 85+ Study, and vegetables were the main source of vitamin A in the LiLACS NZ compared to meats/meat products in the Newcastle 85+ Study, which reflect differences in food availability and dietary choices in the two countries.

### 2.2. Nutritional Status of the Very Old: Example of Micronutrient Deficiency

In the UK, over 10% of older adults and 18% of the very old are at medium or high risk of malnutrition [121]. The British Association for Parenteral and Enteral Nutrition (BAPEN) estimated that the cost of care for disease-related malnutrition will exceed £13 billion per year and over half of that will be expended on older adults [122]. The very old are at increased risk of malnutrition for several reasons, including multimorbidity, polypharmacy, increased hospitalization [123], financial constraints, reduced mobility, social isolation and the loss of independence [124]. These health and social factors are coupled with changes in body composition (i.e., loss of lean mass, increase in fat mass, loss of bone density, and fluid and electrolyte dysregulation), decline in taste sensitivity, poor oral health and malabsorption, as reviewed in [125]. Advancing age and some widely used drugs in this age group have adverse effects on the sense of taste [126,127], on appetite [128] or on nutrient absorption due to drug-nutrient interaction [129]. Although micronutrient malabsorption is not an inherent consequence of ageing, the pH-dependent absorption of vitamins and minerals, such as folate, vitamin B12, calcium, iron and β-carotene, might be partially compromised [130,131]. About 10–30% of older adults have atrophic gastritis which leads to hypochlorhydria [130] and has a detrimental effect on acid-pepsin digestion, resulting in impaired vitamin B12 absorption [132]. Very old adults are also at higher risk of vitamin D deficiency due to reduced skin stores of 7-dehydrocholesterol (provitamin D), which, in combination with reduced sun exposure, leads to less dermal synthesis of vitamin D, renal impairment and reduced renal conversion of the biologically inert to active form (i.e., 25-hydroxyvitamin D (25(OH)D) to calcitriol), immobility, malnutrition and environmental factors (reviewed in [133]). Micronutrient deficiencies contribute to increased disease risk, disability, frailty and impaired physical function in very old adults [134].

Energy requirement declines with advanced age as it follows the decline in physical activity. However, there is currently no convincing evidence that vitamin and mineral requirements decrease as well. The Scientific Advisory Committee on Nutrition (SACN) released energy dietary reference values (DRVs) for the UK in 2011. Energy DRVs were set at 11.5 MJ for men and 9.1 MJ for women aged 25–34, and 9.6 MJ for men and 7.7 MJ for women aged ≥ 75, a decrease of ~2 MJ [135]. However, according to the Committee on Medical Aspects of Food Policy (COMA) 1991 report, most DRVs for micronutrients remain constant throughout adulthood [136]. This difference in requirements between energy and micronutrients adds to the potential risk for nutritional deficiencies (malnutrition) in the very old, as reviewed in [101].

A review of micronutrient deficiencies in community-dwelling older adults (aged ≥ 65) living in developed countries (assessed against the Nordic Nutrition Recommendations estimated average requirement (EAR)) established that more than 20% of older adults had inadequate intakes of vitamin D, folate, calcium and selenium [137]. Similarly, a more recent review concluded that at least 30% of older adults were below the EAR for vitamin D, vitamin B12, calcium, magnesium and selenium [138].

In the UK, the NDNS reported that the intakes of most micronutrients were approximately 10% lower in those aged ≥ 85 than in 65–74-year olds [113]. The results indicated that the vast majority of vitamin and mineral deficiencies increased with age and were dependent on socioeconomic status [139,140]. Specifically, the NDNS rolling programme estimated that 7.3% and 10.8% of older men and women (≥65), respectively, had red blood cell (RBC) folate concentrations < 340 nmol/L, and 5.9% had serum vitamin B12 concentrations < 150 pmol/L [141]. In the Newcastle 85+ Study, there was a relatively low prevalence of ‘inadequate’ folate status (3.6%) [50,98], but a high percentage of participants (17.1%) had plasma vitamin B12 concentration below 148 pmol/L [142].

The 1994–1995 NDNS reported that 13% and 25% of community-dwelling very old men and women, respectively, had 25(OH)D concentrations below 25 nmol/L. These numbers increased to 42% and 35% in institutionalized men and women respectively [143]. In 2005, the Health Survey for England (HSE) reported that 8% of the men and 22% of the women in this age group were considered vitamin D deficient [144]. In an osteoporosis screening trial, 25(OH)D concentrations were measured in 1894 older adults aged ≥ 80 across Europe [145]. Serum 25(OH)D concentrations were lowest (45.7 nmol/L) in Belgium and highest (81.7 nmol/L) in Spain [145]. In the Newcastle 85+ Study, the prevalence of vitamin D deficiency according to the North American Institute of Medicine (IOM) guidelines (serum 25(OH)D < 30 nmol/L) varied significantly with season, with the highest prevalence observed in spring (51%) and the lowest prevalence observed in autumn (23%) [146]. In the LiLACS NZ 85+ study, only 2% had a 25(OH)D concentration < 25 nmol/L, whilst 23% were <50 nmol/L [147].

In summary, very old adults are at an increased risk of malnutrition and micronutrient deficiencies because of a range of biologic and environmental factors, such as multimorbidity, sensory and body composition changes, diminished appetite, decline in oral health, malabsorption, polypharmacy, financial hardships, and social isolation. There is no clear evidence that the requirements for micronutrients decline along with age-related decline in energy intake which exacerbates the issue and highlights the increasing importance of nutrient density in the diets of the very old. Nutrient (and especially micronutrient) requirements of the very old are poorly understood, and there is a major evidence gap that both epidemiological and dietary intervention studies need to address to advance understanding of the relationships between (micro)nutrient intake, status and health outcomes in this age group.

### 2.3. Nutritional Needs of the Very Old: Example of Protein and (Serum) Vitamin D for Muscle Health

As noted above (Section 1.2.1), the protein intake required to sustain muscle mass and function in the very old may be higher than the current RDA of 0.8 g/kg BW/day [83]. The proposed intake of at least 1.0–1.5 g/kg BW/day is based on a limited number of metabolic studies that involved older adults aged ≥ 80 [85,86], and on epidemiological evidence summarized in several expert position papers and reviews (e.g., [51,52,65,66,87,88,89,101]). We confirmed recently that women in the Newcastle 85+ Study who consumed < 1.0 g/kg adjusted BW/day of protein (i.e., BW adjusted for a healthy body mass index (BMI), if outside the range of 22–27 [90]) had lower GS and worse Timed Up-and-Go (TUG) performances at baseline compared with women consuming ≥ 1 g/adjusted BW/day of protein, irrespective of key confounders (e.g., lean mass, multi-morbidity, and physical activity) [53]. However, the subsequent rates of GS and TUG decline over 5 years were not affected by protein intake, and no associations between the current RDA protein cut-off and physical functioning in the very old living in the community were found [53]. We further observed a combined positive effect of protein intake ≥ 1 g/kg adjusted BW/day and higher self-reported physical activity (PA) on muscle strength (i.e., higher GS at baseline and slower GS decline over 5 years compared to inactive participants). However, higher PA was not associated with reduced GS decline in participants with low protein intake (<1 g/kg adjusted BW/day), suggesting that higher PA may be ineffective in the very old if protein intake is not adequate. To our knowledge, this is the only prospective study of the relationship between low protein intake, muscle strength (GS) and physical performance (TUG) in those aged ≥ 85. Data from well-designed and conducted prospective epidemiological and nutritional intervention studies will be needed, to revise the current protein recommendations for muscle function in the very old in relation to a clearly defined clinical outcome (e.g., muscle strength).

Several lines of evidence from epidemiology and intervention studies suggest that vitamin D status influences muscle strength and function (e.g., [148,149,150,151]). Despite mixed results from individual studies, recent systematic reviews and meta-analyses of randomized controlled trials have revealed a small but significant improvement in muscle strength/function in older adults taking vitamin D supplementation, sufficient to raise 25(OH)D concentrations to more than 30 nmol/L [150] or 50 nmol/L [151]. The results from epidemiological studies that included significant numbers of the very old (e.g., [148,149]) have shown a higher risk of poor physical performance if 25(OH)D was <25 nmol/L, but also reported no further improvement in performance at intermediate (>50 nmol/L) and higher concentrations (>75 nmol/L), which suggests that 25 nmol/L may optimize biological function. At a population level, the SACN in the UK recommended that a concentration of 25 nmol/L was a marker of adequacy for general and musculoskeletal health [152], which is much lower than the 50 and 75 nmol/L 25(OH)D inadequacy cut-offs proposed by the Institute of Medicine (IOM, 2011) [153] and the Endocrine Society, USA, respectively [154]. The IOM also recognized the accumulated evidence for a non-linear relationship (a U- or J-shaped curve) between 25(OH)D concentration and non-skeletal outcomes, which has been also reported in the very old for mortality [44] and cognitive function [46]. In the Newcastle 85+ Study, greater longevity and better cognition were observed in those with 25(OH)D concentrations of 40 to 60 nmol/L. Furthermore, muscle strength (GS) declined faster in men in the lowest season-specific quartile of 25(OH)D (the lowest values ranged from 17 to 30 nmol/L), but there was no beneficial effect of being in the highest quartile (the highest values ranged from ≥47 to >69 nmol/L) [47]. Men in both the <25 nmol/L (severely deficient) and the ≥75 nmol/L (sufficient) 25(OH)D group had worse TUG test performances during follow-up compared with those in combined middle categories [47]. The apparently adverse effect of higher 25(OH)D concentrations is poorly understood and may be driven by uncontrolled confounding (e.g., disease related to 25(OH)D deficiency but masked by supplementation) [155]. Future epidemiological and intervention studies are needed to determine the optimum 25(OH)D concentration for muscle function and other clinical outcomes in the very old and to develop the most effective and acceptable strategies (e.g., using both foods and supplements to achieve this optimum).

## 3. Cohorts Dedicated to the Very Old: Additional Findings from the Newcastle 85+ and the LiLACS NZ

Prospective studies using a single birth cohort of the very old (aged ≥ 85) that have conducted a comprehensive assessment of ageing and links with diet are rare [27,28]. Although the very old are included in smaller numbers in other ageing studies [113,114,115,116,117,118], they are often considered to be hard to recruit and difficult to retain in longitudinal research because of the high prevalence of chronic diseases and functional impairments [94,95,156] and supposedly low motivation for research participation. However, the Newcastle 85+ Study has shown that attrition in this cohort during 5 years of follow-up was due mainly to mortality (more than 40%) rather than to withdrawal from the study [157]. In addition, we have found that the repeated 24-h recall is a feasible retrospective dietary method in this age group [112]. Similar multidimensional health assessments and dietary assessment approaches were used to investigate health and functioning trajectories in very old Māori and non-Māori participants in the Newcastle 85+ Study sister study, the LiLACS NZ [28]. In this section we summarize briefly the main characteristics of participants and update the recent findings [100] from nutritional epidemiological investigations in both studies.

### 3.1. The Newcastle 85+ Study

The Newcastle 85+ Study is a prospective, population-based study of a single birth cohort (born in 1921) that was recruited in 2006 through general practices in Newcastle and North Tyneside area of North East England, UK. At baseline, 845 participants had complete multidimensional health assessments and medical record reviews. Participants were followed up at 1.5 (wave 1), 3 (wave 2), and 5 years (wave 4) [27]. Complete dietary data (two-day 24-h multiple pass recall (24-h MPR) [112]) without protocol violations were available for 793 participants (302 men and 491 women) at baseline. Details about the study protocols and questionnaires used at each wave can be found at http://research.ncl.ac.uk/85plus/. To investigate the relationship between health outcomes (e.g., muscle strength, physical performance and cognitive function) in the very old, both dietary pattern (whole diet) and single nutrient approaches were used.

#### 3.1.1. Dietary Assessment in the Very Old

Prior validation of the 24-h MPR in a subsample of this cohort (*n* = 89) revealed that, compared with the EPIC FFQ (i.e., frequency of intake of 134 foods in the past year) [114], this dietary assessment method provides more accurate estimates of intakes of energy and nutrients, and that is an acceptable approach for use in the very old [112]. At baseline (wave 1), trained research nurses recorded detailed food intakes (paper-and-pencil) on two non-consecutive days of the week, at least one week apart. No recalls were conducted on Saturdays and Sundays.

Portion sizes of foods and drinks were estimated from a standardized photographic atlas [158] and food packages. Dietary data were coded based on McCance and Widdowson’s, “The Composition of Food, 6th edition” [159], and double-entered into a Microsoft Access data base. All discrepancies between the two data bases were resolved and checked against original dietary records before data analyses. Individual foods were grouped into 118 distinct groups (based on their similarities and nutrient content) established by the Human Nutrition Research Centre, Newcastle University. These food groups were further collapsed into 33 groups, and 30 were used in the cluster analysis to derive dietary patterns (DP) [160].

### 3.2. Dietary Patterns and Physical Functioning in the Very Old

The main advantage of DP analysis in investigating the relationship between diet and health is the ability to take into account the complexity of human diet and food interactions within a whole diet. DP may be derived using statistical methods (e.g., factor and cluster analysis), a data-driven (a posteriori) approach that does not rely on the prior knowledge about the diet–health relationship [161]. A more common approach for deriving DP is a hypothesis-driven (a priori) approach which is based on dietary indices for a specific diet (e.g., Mediterranean-style diet) or dietary guidelines for a healthy DP (e.g., Healthy Eating Index) [162]. The latter approach is reliant on current scientific evidence about healthy diets which may not be accurate for the very old and is limited in its understanding of the effect of whole diet on health.

Using data for intakes (yes/no) of each of the 30 food groups, we established three distinct dietary patterns (‘High Red Meat’, ‘Low Red Meat’, and ‘High Butter’) that varied with key sociodemographics (e.g., education and, social class), health and functioning measures (e.g., disability, cognitive impairment, and physical activity). Eight food groups contributed the most to DP separation (e.g., butter, unsaturated fats spreads/oils, gravy, potato/potato products, red meats/meat products, and legumes). DP1 (‘High Red Meat’) had a high proportion of participants consuming meats, potatoes, unsaturated fat spreads, and a low proportion of those eating butter. DP2 (‘Low Meat’) was under-represented by those eating meats, potato, and gravy but had the highest proportion of participants eating fruits, nuts, whole grains, fish/sea food, dairy, soups, coffee and alcohol. This DP was considered to be the healthiest and was used as a reference in the analyses. DP3 (‘High Butter’) had the highest proportion of participants eating butter and the lowest proportion of those eating unsaturated and saturated fat spreads [160]. Compared with others, participants in DP2 (‘Low Meat”) were more educated, belonged to a higher social class, lived in more affluent areas, were more physically active, and were less likely to have a cognitive impairment or disability, or to be obese [160].

When tested as a main effect in mixed models, participants in DP1 (‘High Red Meat’) had a mean GS of 0.92 kg lower and men had a GS 0.92 kg lower (both *p* = 0.05) compared with those in DP2, after adjustment for sociodemographic, lifestyle, health factors and total energy. Men in DP3 (‘High Butter’) had a steeper loss of GS (mean 0.63 kg per wave) over 5 years compared to men in DP2 (*p* = 0.05). In the fully adjusted model, participation in DP3 (‘High Butter’) was associated with an overall longer time needed to complete the TUG test compared with DP2 (*p* = 0.002), and a faster rate of linear decline over 5 years across the entire cohort (*p* = 0.04). In addition, men in DP1 (‘High Red Meat’) and women in DP3 (‘High Butter’) had worse TUG performances at baseline compared to those in DP2, but the rate of decline did not vary by DP group [163].

Participants in DP3 (‘High Butter’) had the highest total fat and % energy from fat, cholesterol, and saturated fatty acids (SFA), and % energy from SFA [160], which may have compromised ageing muscle fibres by increasing insulin resistance, inflammation and intramyocellular lipid deposits, and diminishing muscle quality [164,165].

### 3.3. Nutritional Biomarkers and Physical Functioning in the Very Old: 25(OH)D

Because of seasonal variation in serum 25(OH)D, which are apparent in the very old as in other age groups (see Section 2.2) [146], we used season-specific 25(OH)D quartiles, which is a preferred method to account for the cyclic nature of vitamin D [166,167]. Trajectories of muscle strength (GS) and physical performance (TUG) differed by sex [47], and the intake of vitamin D in supplements and in medication (e.g., prescription vitamin D, calcium with vitamin D, bisphosphonate with vitamin D and calcium) was an important effect modifier of cognition [46] and longevity [44] in the very old. Separate analyses of the relationship between 25(OH)D and muscle function were conducted in the participants not taking vitamin D supplements or medication (*n* = 678).

Participants in the highest season-specific quartile (SQ1) [44,46] were more likely to be women, to take vitamin D supplementation/medicine, and to have higher risk of cognitive decline [46] and mortality [44], compared with those in combined middle quartiles (SQ2 + SQ3). When examined as a main effect in the mixed models, we observed a U-shaped relationship, with lower muscle strength (GS) at baseline in all participants—men and women—in both SQ1 and SQ4 compared with SQ2 + SQ3. For example, SQ1 was associated with a 2.56 kg (*p* = 0.008) and SQ4 with a 2.16 kg (*p* = 0.04) lower GS in men, compared to those in the middle quartiles, and men in SQ1 experienced a 0.44 kg (*p* < 0.001) decline in GS annually over 5 years of follow-up. In participants not taking vitamin D supplements/medication, SQ1 was associated with lower GS and GS declines over the study period. After adjustments for key covariates, only SQ1 was associated with a GS decline (1.41 kg annually, *p* = 0.003) in men, in all participants and in those who were not supplemented with vitamin D, but not in women [47].

Similarly, we observed a U-shaped relationship between SQ1 and SQ4 and physical performance (TUG) at baseline in all participants and in women, compared to those in the middle quartiles. However, after adjustments for key covariates (e.g., anthropometry, health variables, and use of walking aids) the U-shaped relationship remained only in women. However, the rate of decline in TUG did not vary across the 25(OH)D quartiles over 5 years in all participants or in men and women.

The results from prospective studies that investigated the change in muscle function in relation to 25(OH)D in older adults and in those aged ≥ 85 need further evaluation [47,148,149,150,151,168]. Most have hypothesized a protective effect of higher 25(OH)D concentrations (≥50 or ≥75 nmol/L) and have found an increased risk of decline if 25(OH) is below the 30 or 50 nmol/L cut-offs [148,149], but others have shown no effect [168,169]. Comparisons of the results from these studies that included the very old are challenging because of differences in definitions of suboptimal levels of 25(OH)D for physical functioning, the use of different tests to assess physical performance, duration of follow-up, and selection of confounding factors. Maintaining serum 25(OH)D at a concentration of 40 to 60 nmol/L through a healthy diet rich in vitamin D (e.g., oily fish and eggs) or through supplementation [166] may be beneficial for muscle function in very late life.

### 3.4. Prevalence and Determinants of Low Protein Intake in the Newcastle 85+ Study

The amount of protein that the very old get from their diet, the foods that provide most protein and the amounts of protein eaten at different times of the day are all factors that may play important roles in delaying, or possibly preventing, protein malnutrition and associated diseases, but the evidence base in this area remains fragmentary. Such information is essential for developing new food products and public health policies to better tackle malnutrition. We investigated the prevalence and determinants of low protein intake in 722 community-dwelling, very old adults, participating in the Newcastle 85+ Study [99]. Low protein intake was defined as an intake < 0.8 g of protein per adjusted BW/day [90]. Twenty eight percent (*n* = 199) of the community-living, very old participants in the Newcastle 85+ Study had low protein intakes. Participants were less likely to have low protein intake when meat and meat products had higher percent contribution to the total protein intake (OR: 0.97, 95% CI: 0.95, 1.00), but more likely to have low protein intake if cereal and cereal products and non-alcoholic beverages made a higher percent contribution to the total protein intake. Morning eating occasions contributed more to the total protein intake in the low (<0.8 g/kg adjusted BW/day) compared to the adequate protein intake group (≥0.8 g/kg adjusted BW/day; *p* < 0.001). Being a woman (*p* < 0.001), having higher energy intake (*p* < 0.001) and having higher tooth count (*p* = 0.047) were associated with higher protein intakes in adjusted models. This study provided evidence that low protein intake is prevalent in the very old. In addition, it provided information on protein intake patterns and food group contributors to protein intake, which could be helpful when considering interventions for improving protein intake in this fast-growing population group [99].

### 3.5. Life and Living in Advanced Age Study (the LiLACS Study): A Cohort Study in New Zealand-e Puãwaitanga o Nga Tapuwae Kia Ora Tonu

The LiLACS NZ study [28] filled a gap in knowledge about the nutritional status and health of the very old Māori and non-Māori communities. In 2010, the study recruited 421 Māori participants born in 1920–1930, and 516 non-Māori participants born in 1925. At the 12-month multidimensional health follow-up assessment in 2011, 216 Māori and 362 non-Māori participants completed the two-day 24-h MPR [119]. Interviewers for Māori were fluent in Māori language and culture. Because of cultural preferences, foods consumed by older Māori differ from those of non-Māori older adults which may result in differences in intakes of nutrients and other food constituents.

#### 3.5.1. Dietary Assessment in the Very Old

The 24-h MPR was conducted on two different days of the week which were, on average, 17 to 23 days apart. Food and drink portion sizes were recorded from food packets and labels, or estimated using the same photographic atlas used in the Newcastle 85+ Study [158] and adapted for the LiLACS study. The New Zealand Food Composition Data Base (NZCDB) [170] and FOODfiles (an electronic subset of the NZCDB) were used to code foods and to calculate energy and nutrient intakes. Food codes were further combined into the 33 food groups used in the 2008/07 New Zealand Adult Nutritional Survey (NZANS).

### 3.6. Food Contribution to Macronutrients: Comparison between Very Old Māori and Non-Māori Adults

In non-Māori, the median energy intake was 7.91 MJ/d in men and 6.26 MJ/d in women, with 45% derived from carbohydrates, 36.7% from fat, and 15.4% from protein. Compared with Māori, non-Māori older adults had higher intakes of energy (Table 2), alcohol, dietary fibre and total sugars, but less energy-adjusted protein [119].

Dietary fibre intake was relatively low in both ethnic groups (median of 18.2 and 21.7 g, respectively) compared with current recommendations [171]. For both population groups, bread was the main contributor to energy and carbohydrates. Meats (beef, veal, and poultry), fish/sea food, and milk contributed the most to protein intake with some differences in the order of top contributors by ethnicity and sex. Butter and margarine were the main sources of fat for all participants. In Māori, the acceptable macronutrient distribution range (AMDR) [172] for protein was met by 39% of women and 36% of men and the AMDR for fat was met by all. However, the adequate intake (AI) [172] for water was met by only 11% of women and 4% of men. In non-Māori older adults, about 45% of men and women met the AMDR for protein, and only 11% (women) and 2% (men) met the AI for water [119].

A high prevalence of malnutrition (assessed by the Seniors in the Community: Risk Evaluation for Eating and Nutrition questionnaire) was observed in both Māori (49%) and non-Māori (38%) older adults [173], suggesting inadequacies for a range of nutrients.

### 3.7. Food Contribution to Micronutrients: Comparison between Very Old Māori and Non-Māori Adults

Similar to non-Māori (see Table 2; Section 2.2), in Māori participants, vegetables were the main source of vitamin A (42% for women and 35% for men). Cereals (grains), bread, and vegetables were the main contributors to folate intake. The main three foods contributing to vitamin D intake were milk (about 26%), followed by butter/margarine, and fish/sea food. Milk was also the main source of calcium (for 33% men and 34% women), followed by bread (in women). Meats and bread were the main sources of zinc in both sexes [120].

The national nutritional survey, the NZNS, has limited data on the micronutrient status of the very old. Non-Māori, very old adults are routinely combined with those aged ≥ 70, and Māori, with adults aged ≥ 50. This poses a challenge in determining nutritional status in, and nutritional requirements of, the very old in New Zealand, and so the LiLACS NZ study represents a valuable source for comprehensive analyses of nutrition in advanced age. Future studies will investigate dietary intakes in relation to nutritional biomarkers and health outcomes in Māori and non-Māori, very old adults.

## 4. Challenges in Establishing Nutritional Needs in the Very Old

There are several challenges related to collection of dietary data in the very old, such as inability of older adults to accurately recall dietary information due to cognitive and functional impairments. Heterogeneity in health and function and the presence of multiple diseases (multimorbidity) that require various and complex treatments in later life pose additional challenge in nutritional research that tries to elucidate the relationships between diet (nutrients), single diseases and functioning in the very old.

### 4.1. Challenges with Nutritional Assessments in the Very Old

To have an accurate record of the habitual food intake of an individual or group of individuals, and to understand nutrition-related outcomes, collection of robust dietary intake data is vital. Dietary assessment at any life stage presents challenges [110]. In the future, the development of biomarkers of dietary exposure may reduce or eliminate the need for subjective self-report, but meanwhile, dietary assessment remains labour intensive and costly. Methods at researchers’ disposal include weighed dietary intakes, estimated weight food diaries, food records and FFQ. Each method requires varying levels of commitment, time and cognitive ability from the respondent, as well as the researcher’s time and skill. The choice of dietary assessment depends on the research question, and on the population group to be assessed. Assessing food choice and/or nutrient intake in older people, particularly in the very old, is challenging for several reasons. The respondent may have little or no involvement in food purchasing or preparation, cognitive impairment may restrict his/her ability to recall intake, and ability to record intake may be limited by physical limitations, sensory impairment and communication difficulties. The interviewer may need to rely on one or several carers as a proxy reporter of dietary intake, which increases the probability of errors and misreporting. Thus, in this age group, it is important that the chosen retrospective dietary assessment method is not dependent on self-recording of intake by participants per se.

### 4.2. Heterogeneity in Health in the Very Old

The very old are a very heterogeneous population group ranging from healthy and active individuals with few disabilities to those with multiple diseases (multimorbidity) [94,95,96,156]. In the baseline assessment of the Newcastle 85+ Study, no individual was completely free of chronic diseases and the median number of diseases was five (IQR: of 3–6) [156]. This heterogeneity leads to both practical and conceptual difficulties in determining the nutritional needs of the very old and in making appropriate public health recommendations. For example, there is limited understanding and agreement of the outcome measures that should be used to derive nutritional guidelines and nutritional adequacy in this age group. Most physical and cognitive functions decline with age, but the great inter-individual variability in the age of onset and how rapidly the decline progresses creates difficulties (but also opportunities) in assessing biomarkers of healthy ageing [174,175]. Furthermore, the relationships between biomarkers and health outcomes are frequently considerably different between the very old and the young-old. For example, whilst hypertension is a well-established risk factor for CVD and mortality among younger adults, the same relationship is not observed in those aged ≥ 85 [176].

The evidence is scarce regarding the relationship between nutrients (diet), health and functional outcomes in the very old [44,45,46,47,50,53]. The current guidelines for nutrients, such as, for example, protein [83] (IOM, 2005) and vitamin D [152] (SACN, 2016) for muscle health, do not differ across age groups, activity levels, body composition, disability, multimorbidity or outcome measures (e.g., muscle mass, muscle strength). Because of their complex health needs that require multiple medications, decreased activity and energy levels, and age-related changes in the musculoskeletal and gastrointestinal systems, dietary requirements in the very old aimed at ameliorating functional decline and supporting healthy ageing need further systematic investigation to test the assumption that recommendations used in younger age groups are also applicable in this age group.

Furthermore, because energy intakes of those aged ≥ 85 are, typically, about one third lower than those in younger adults, there are particular challenges in achieving adequate intakes of protein, essential fatty acids and micronutrients in the much smaller amounts of foods consumed at this life stage. This is likely to mean that the nutrient density of the whole diet will need to increase substantially in later life which posits a translational challenge with respect to the necessary changes in food habits and preferences. There may be opportunities to develop attractive new food products which are higher in protein and/or other nutrients specifically for this population group, and there may be benefits from restricting the intakes of higher sugar and higher fat food products which have relatively low nutrient densities. For other (younger) population groups, the use of nutritional supplements is discouraged (because of the lack of evidence of benefit and evidence of possible hazards from higher intakes of some micronutrients) [177]. However, for the very old, it will be important to revisit this issue and to consider the feasibility, acceptability, efficacy and safety of using food supplements and/or food micronutrient enrichment to ensure adequate intakes by this vulnerable population group.

## 5. Conclusions

Dietary assessment in very old adults is challenging because of the higher prevalence of cognitive and physical impairment and reliance on proxy reporting in this age group. However, several European studies of ageing, and two specialized cohorts of the very old have successfully collected nutritional data on representative population samples. In the Newcastle 85+ Study and the LiLACS NZ Study, the two-day 24-h MPR was an acceptable retrospective method for estimating intakes of the whole diet at the individual level. Comparisons of energy and nutrient intakes between the studies should be interpreted with caution because of the differences in dietary assessment methodology, nutrient composition tables, and participants’ age and health statuses. Comparable results were obtained in the studies that used the same methodology (e.g., the Newcastle 85+ Study and the LiLACS NZ study). Very old adults have high risks of macronutrient malnutrition (e.g., low protein intake) and micronutrient deficiencies (e.g., vitamin D, calcium and magnesium). Carbohydrates were the main source of energy, and cereals/cereal products and bread were the main contributors to intakes of energy and most macronutrients, folate and iron. Meats and milk were the major dietary sources of protein and also of vitamin B12.

The very old may require higher protein intake, >1 g/kg BW/day, in combination with exercise (PA) to sustain muscle function. Maintaining serum 25(OH)D concentrations between 40 and 60 nmol/L may be beneficial for musculoskeletal health. Dietary patterns characterized by higher intakes of fruits, vegetables, nuts, dairy, fish and whole grains may delay muscle strength decline. These results need to be repeated and corroborated in other prospective studies of the very old.

Despite the increasing epidemiological evidence for the role of diet (nutrients) on heath and functioning, the nutritional requirements of older adults are poorly understood. The evidence is particularly limited in the very old and there is not yet any consensus on the conceptual approaches which should be used when establishing dietary requirements of this highly heterogeneous population group. For example, the use of appropriate health outcomes (such as muscle strength and physical and cognitive performance) against which to assess nutritional requirements need to be identified and defined clearly, as do procedures for addressing inter-individual nutritional needs associated with the very common, but heterogeneous, manifestations of multimorbidity and polypharmacy in later life.

## Figures and Tables

**Table 1:** Characteristics of the European studies including the very old and the very old specialized cohorts *.

Study	Study Characteristics and Population	Dietary Assessment	Other Assessments
NDNS 65+	Carried out during 1994–1995 in the UK; included two nationally-representative samples of adults aged ≥ 65 (community-dwelling and living in institutions); 459 (172 men and 287 women) aged ≥ 85 completed four-day weighted diet record [113].	Four-day weighted diet records	Health background questionnaire and blood samples
EPIC-Oxford	Started in 1993 in Oxford, UK; designed to investigate the relationship between diet and cancer; 1283 (411 men and 872 women) aged ≥ 80 completed the FFQ by the third follow-up (2010–2014) [114].	FFQ	Health and lifestyle questionnaire, and blood samples
DNFCS	Conducted in the Netherlands in 2010–2012; included nationally-representative sample of older adults aged ≥ 70; 225 (103 men and 122 women) aged ≥ 80 completed both 24-h dietary recall [115].	Two 24-h dietary recall	Heath background questionnaires anthropometric measures
InCHIANTI	Conducted in 1998 in Tuscany, Italy; included participants aged 21 to 103; 1436 completed the FFQ; 170 (60 men and 113 women) were aged ≥ 85 and had dietary data [116].	FFQ	Background questionnaire (sociodemographic, lifestyle, function)
GNS	German nationally-representative study of community-dwelling older adults; conducted on behalf of the German Ministry of Health in 1998; 287 (89 men and 198 women) aged ≥ 85 had complete dietary data [117].	Three-day dietary records	Background questionnaire (sociodemographic, lifestyle)
ANS	Austrian nationally-representative sample of older adults; survey conducted in 2003 had 115 older adults aged ≥ 85 (22 men and 93 women) [118].	Three-day dietary records	Heath background questionnaire
NC85+ *	A longitudinal, population-based study; recruited over 1000 participants aged 85 from Newcastle and North Tyneside, UK in 2006; 845 (319 men and 526 women) had complete multidimensional health assessment and medical records review; 793 (302 men and 491 women) had complete dietary data [97,98].	Two 24-h dietary recall	Multidimensional health and functioning assessment; medical records review
LiLACS NZ *	Population-based cohort study of 937 very old adults from the Bay of Plenty and Rotorua region, New Zealand, recruited in 2010 (421 Māori aged 80–90 and 516 non-Maori participants aged ≥ 85); 216 Maori (92 men and 124 women) and 362 non-Māori (172 men and 190 women) had complete dietary data [119,120].	Two 24-h dietary recall	Background health and functioning questionnaire; blood samples

NDNS 65+, National Diet and Nutrition Survey of people aged ≥ 65; EPIC, European Prospective Investigation into Cancer and Nutrition; DNFCS, Dutch National Food Consumption Survey; InCHIANTI, Invecchiare in Chianti, Aging in the Chianti Area; GNS, German Nutrition Survey; ANS, Austrian Nutrition Survey; NC85+, Newcastle 85+ Study; LiLACS NZ, Life and Living in Advanced Age New Zealand: Te Puãwaitanga o Nga Tapuwae Kia Ora Tonu. FFQ, food frequency questionnaire. * Specialized cohorts of the very old.

**Table 2:** Energy and nutrient intake in European and European descendent older adults aged ≥ 80 and ≥85 ^1^.

Cohort				Men										Women						
	Energy	Carb	Fat	Protein	Fibre	Folate	B12	D	Ca	Iron	Energy	Carb	Fat	Protein	Fibre	Folate	B12	D	Ca	Iron
	MJ/d	%	%	%	g/day	μg/day	μg/day	μg/day	mg/day	mg/day	MJ/day	%	%	%	g/day	μg/day	μg/day	μg/day	mg/d	mg/d
NDNS 65+	6.99 ^3^	48.5	36.3	15.2	11.4 ^4^	219	3.8	2.8	717	9.7	5.60 ^3^	48.4	36.8	14.5	9.4 ^4^	170	2.9	2.0	619	7.5
EPIC ^1^	9.84	49.7	31.4	15.5	24.5 ^4^	466	7.5	4.2	1157	18.1	9.02	50.3	31.5	16.3	24.0 ^4^	461	7.5	4.0	1147	17.0
DNFCS	7.40	41.4	34.0	16.4	20.0	46 ^5^	4.9	3.9	1016	9.6	7.30	41.0	35.0	15.6	16.2	34 ^5^	4.4	2.9	2030	8.3
InCHIANTI ^2^	7.38	50.0	29.0	16.0	17.2	228	-	-	778	11.5	6.36	50.0	32.0	16.0	15.3	200	-	-	701	9.6
GNS	9.34	44.2	33.2	16.3	23.7	123 ^6^	-	3.8	721	13.3	8.07	42.6	35.0	16.2	19.9	106 ^6^	-	2.7	729	12.6
ANS	7.40	44.0	40.0	14.0	15.0	174 ^6^	4.0	3.4	642	10.0	7.10	43.0	40.0	16.0	16.0	166 ^6^	3.9	3.1	649	11.1
NC85+	7.73 ^3^	46.8	36.4	15.9	11.3 ^4^	245	3.4	2.3	829	10.5	6.15 ^3^	46.8	37.2	15.5	9.3 ^4^	189	2.6	1.8	683	7.8
LiLACS NZ ^7^	7.90 ^3^	44.3	36.2	15.6	22.8	245	3.6	4.1	731	11.6	6.27 ^3^	46.4	37.2	15.3	20.4	215	2.6	3.4	679	9.3

^1^ Adapted from Hill et al. [100]. Values are medians unless indicated otherwise. NDNS 65+, National Diet and Nutrition Survey of people aged ≥ 65 [113]; EPIC, European Prospective Investigation into Cancer and Nutrition [114]; DNFCS, Dutch National Food Consumption Survey [115]; InCHIANTI, Aging in the Chianti Area [116]; GNS, German Nutrition Survey [117]; ANS, Austrian Nutrition Survey [118]; NC85+, Newcastle 85+ Study [97,98]; LiLACS NZ, Life and Living in Advanced Age New Zealand: Te Puãwaitanga o Nga Tapuwae Kia Ora Tonu [119,120]; Carb, carbohydrates; B12, vitamin B12; D, vitamin D; Ca, calcium; -, not available. ^2^ Values are means. ^3^ Without alcohol intake. ^4^ Non-starch polysaccharides (NSP). ^5^ Only folic acid. ^6^ Dietary folate equivalents: 1 µg DFE = 1 µg food folate = 0.5 µg folic acid supplement (fasting) = 0.6 µg folic acid from fortified food or as supplement (non-fasting). ^7^ Non-Māori participants (European descendants).
